# Self-regulated vocal health in undergraduate vocal music education: a qualitative needs analysis

**DOI:** 10.3389/fpsyg.2026.1894233

**Published:** 2026-08-05

**Authors:** Guo Xiao, Chamil Arkhasa Nikko Mazlan

**Affiliations:** 1 Department of Music and Music Education, Faculty of Music and Performing Arts, Sultan Idris Education University, Tanjong Malim, Malaysia; 2 Faculty of Music, National Academy of Arts, Culture and Heritage, Kuala Lumpur, Malaysia

**Keywords:** educational psychology, learner agency, needs analysis, performance psychology, self-regulated learning, undergraduate singers, vocal health, vocal music education

## Abstract

Self-regulated vocal health is an important but underdeveloped dimension of undergraduate vocal music education. While vocal training commonly emphasizes technique, repertoire, interpretation, and performance competence, less empirical attention has been given to how students learn to monitor vocal condition, regulate practice behavior, manage emotional readiness, and protect the voice across daily learning and performance preparation. This qualitative needs analysis examined how vocal health self-regulation is understood, represented, and required within undergraduate vocal music education. Drawing on document mapping of ten selected vocal music textbooks and semi-structured interviews with five vocal music education experts, the study examined contextual issues in vocal music education, expert-identified vocal health needs, and the target needs and learning needs required to support students’ self-regulated vocal development. The results indicate that vocal health is widely recognized as essential for learning continuity, performance quality, psychological confidence, and long-term vocal sustainability. However, selected instructional materials and expert accounts gave limited systematic attention to self-monitoring, preventive behavior, rest management, anxiety regulation, and decision-making during practice. Experts associated students’ vocal fatigue, hoarseness, strain, and misuse with incorrect technique, excessive practice, insufficient rest, weak self-protection awareness, performance anxiety, and limited metacognitive monitoring. The needs analysis identified vocal health as a necessary learning domain, current material and behavioral gaps as key lacks, systematic and practical resources as expert-recommended wants, and self-monitoring, behavioral regulation, emotional readiness, and sustainable practice as central learning needs. The study contributes to educational psychology and performance-related music education by identifying expert-informed psychological learning needs related to vocal health self-regulation. Rather than demonstrating psychological processes empirically, the findings provide a needs-based foundation for conceptualizing vocal health as a learning domain involving awareness, self-monitoring, behavioral regulation, emotional readiness, and sustainable vocal practice.

## 1 Introduction

Undergraduate vocal music education is commonly organized around the development of vocal technique, breath control, repertoire mastery, interpretation, and performance competence, as reflected in major vocal pedagogy materials used in Chinese higher education (Yang, 2007; Cao, 2010; Jin, 2013; Xu and Cui, 2013). While these dimensions remain central to vocal training, they do not fully address the psychological and behavioral demands involved in sustaining healthy voice use over time. Unlike many other musical instruments, the singing voice is inseparable from the body, emotion, health behavior, and self-perception of the learner. This is particularly important because music students’ health behaviors have been associated with health responsibility, self-efficacy, affective state, and self-regulation (Ginsborg et al., 2009), while vocal learning and performance are also shaped by confidence, anxiety, self-efficacy, and motivation (Hu, 2024; Pan et al., 2025). Vocal learning is therefore not only a technical or artistic process, but also a self-regulatory process in which students must learn to monitor vocal condition, recognize fatigue or strain, regulate practice intensity, manage emotional tension, and adopt preventive vocal care behaviors (Zimmerman, 2015; McPherson et al., 2017; Sezin et al., 2025).

Recent research on musicians’ health education shows that performance-related health problems remain a concern among music students and teachers. Evans et al. (2024) argued that musicians’ health education should be embedded within music training and should address not only knowledge transmission, but also behavior change, injury prevention, and long-term adoption of healthy practice strategies. This is consistent with wider music psychology research showing that higher education music students’ health attitudes, stress management, and health responsibility require explicit pedagogical attention (Araújo et al., 2017), and that musicians’ health courses can increase students’ knowledge and skills concerning health risks and protective measures (Rosset et al., 2022). This is particularly relevant to undergraduate vocal training, where students may acquire technical skills without systematic guidance on how to protect, monitor, and regulate their voices in daily learning and performance contexts.

The need for more systematic vocal health education is also supported by studies of singers, preservice music teachers, and professional voice users. Yuksel et al. (2025) found that high school choir singers demonstrated poor voice hygiene and limited voice-health knowledge, suggesting that singers may engage in vocal activity without adequate understanding of preventive care. Brunkan (2018) similarly reported that preservice music teachers experienced vocal fatigue and changes in vocal health during student teaching, partly linked to overuse and increased vocal demands. Flynn (2020) further observed that vocal health education in undergraduate performing arts training can be inconsistent, with students receiving limited systematic knowledge of vocal injury and voice disorders. Although these studies focus on different populations, they point to a common issue: vocal health problems often emerge when vocal demands increase but learners’ knowledge, habits, and self-monitoring strategies remain underdeveloped.

Intervention-oriented studies further suggest that vocal health education can improve awareness and self-management, although the evidence remains uneven across populations and contexts. Liddell et al. (2026) found that an online vocal health education programme for music therapists improved self-perceived knowledge, confidence, awareness, and reported vocal health practices. Lücking and Claßen (2026) showed that preventive voice training can have positive effects on vocal function, vocal self-concept, and wellbeing among university teachers and academic advisers. In vocal pedagogy, Diaz et al. (2026) reported preliminary positive findings from flow phonation voice exercises for undergraduate singers in training. These studies suggest the potential value of preventive and educational approaches, but they do not directly explain what undergraduate vocal students need in order to develop vocal health self-regulation within their own pedagogical context.

Self-regulated learning provides a useful psychological framework for understanding this issue because it conceptualizes learners as active agents in their own learning processes. Zimmerman (2015) defines self-regulated learning as a self-directive process through which learners transform their mental abilities into task-related skills. This process involves goal setting, monitoring, strategy use, self-evaluation, motivation, and adaptive control. In vocal music education, these processes are especially relevant because students must not only develop vocal technique and musical interpretation, but also regulate practice habits, vocal condition, emotional state, and long-term health behaviors. McPherson et al. (2017) similarly argue that self-regulated learning supports musical skill acquisition by helping students become more autonomous, reflective, and strategic in practice. For singers, this means making continuous decisions about practice intensity, vocal fatigue, technique use, rest, recovery, and help-seeking. Vocal learning therefore requires not only teacher-led correction, but also students’ active capacity to monitor and adjust their own vocal behaviors.

Within voice-related research, self-regulation is increasingly recognized as central to vocal health management. Sezin et al. (2025) developed and validated the Vocal Health Daily Tracking Form to monitor daily compliance with vocal hygiene practices and home-based voice therapy exercises. Although their work was situated in voice therapy and rehabilitation, its implications extend to vocal education because it highlights daily tracking, self-report, motivation, compliance, and behavioral management as important components of vocal health. For undergraduate vocal students, similar self-regulatory processes may be needed before voice problems become severe, particularly in relation to recognizing vocal fatigue, regulating practice habits, maintaining hydration, applying vocal hygiene strategies, and seeking support when vocal discomfort occurs.

Self-regulated learning research in music education also supports this direction. Zarza-Alzugaray et al. (2025) showed that self-regulated learning among conservatory students is shaped by teacher support, practice hours, music performance anxiety, helplessness, and perceived independence. This indicates that self-regulation in music learning is not simply an individual trait, but a socially and emotionally shaped process. Peistaraite and Clark (2020) also showed that emotion regulation can benefit self-regulated learning among classical musicians. For vocal students, whose learning is closely connected to bodily awareness, emotional expression, confidence, and performance pressure, vocal health self-regulation should be understood as a multidimensional construct involving knowledge, behavior, metacognition, emotion, and pedagogical support.

The psychological dimension of vocal learning further strengthens this argument. Studies on mental regulation, performance anxiety, and vocal therapy suggest that vocal learning is influenced by emotional states, anxiety, self-emotional regulation, coping strategies, resilience, and psychological readiness (He, 2020; Vasylevska-Skupa et al., 2024; Zhang, 2022). Hu (2024) also showed that self-efficacy and motivation were negatively associated with music performance anxiety. These findings indicate that vocal education cannot be separated from students’ psychological experiences, especially when vocal health, performance pressure, and sustainable learning are concerned. Students may know that they should rest, hydrate, warm up, or avoid vocal misuse, but their actual behavior may still be shaped by anxiety, motivation, confidence, teacher expectations, performance pressure, and peer comparison. A psychologically informed approach to vocal health education must therefore consider how students think, feel, monitor, and regulate themselves within the learning environment.

Despite these developments, existing research has often examined musicians’ health education, voice hygiene knowledge, preventive voice training, vocal therapy, music performance anxiety, and self-regulated learning as separate areas. Less attention has been given to the specific needs that should guide vocal health self-regulation within undergraduate vocal music education, especially in contexts where vocal training remains strongly technique centered. This gap is particularly visible in China. Guo and Mazlan’s (2024) scoping review of vocal health literature in China found that vocal health remains insufficiently addressed in current curricula despite its importance for performance enhancement and injury prevention. Their review also showed that existing studies are concentrated largely in medical fields, indicating the need for more interdisciplinary integration between vocal pedagogy, health education, and preventive vocal care.

Needs analysis offers a useful approach for addressing this gap because it can clarify not only what is missing, but also what learners require, what experts perceive as necessary, and what forms of pedagogical support may be needed. In educational and training contexts, needs analysis is commonly used as an early stage before planning, design, implementation, and evaluation. Gould et al. (2004) describe training needs analysis as the first stage of a cyclical educational process that begins with consultation and diagnosis before moving toward course planning and evaluation. Conceptually, needs analysis is multidimensional. Bocanegra-Valle (2016) explains that needs analysis may include necessities, lacks, wants, goals, difficulties, preferences, expectations, and constraints. This is useful for the present study because vocal health self-regulation cannot be understood only as a lack of textbook content. It may also involve missing behavioral strategies, limited psychological readiness, insufficient pedagogical support, and weak integration between vocal technique and health education.

Recent needs assessment research also emphasizes the importance of using multiple sources. Choi and Park (2024) found that many learning needs assessment studies relied mainly on learner-only data and recommended more multifaceted approaches involving diverse stakeholders and complementary indicators. This supports the present study’s use of selected vocal music textbooks, relevant literature, and expert interviews. In music education, Pu et al. (2025) demonstrated how needs analysis can identify target needs and learning needs in higher education music contexts. Their study shows that needs analysis can clarify what learners require, what they lack, what they want, and what pedagogical routes may support future development. Needs analysis is also useful for designing learning materials that are responsive to learners and stakeholders (Anwar et al., 2023; Mat et al., 2025). Adapting this logic to vocal health education, the present study conceptualizes vocal health self-regulation as a target learning need in undergraduate vocal training.

The rationale for the present study is summarized in Table 1. Rather than treating vocal health as a general pedagogical concern, the table maps the literature-based problem areas to the specific needs addressed by this qualitative needs analysis. This mapping clarifies why the study focuses on contextual issues, expert-identified needs, and the target and learning needs required to support vocal health self-regulation.

**TABLE 1:** Problem–need–research question alignment for the present study

Problem area	Evidence from literature	Critical gap identified	Need for the present study	Related RQ
Vocal training remains strongly technique-centered, while vocal health is less systematically embedded.	Musicians’ health education should be embedded within music training and should address behavior change, injury prevention, and long-term adoption of healthy strategies (Evans et al., 2024). Vocal health education in undergraduate performing arts training is often inconsistent, and students may receive limited systematic knowledge of vocal injury and voice disorders (Flynn, 2020).	Existing training may develop technique and repertoire competence without equally developing students’ capacity to protect, monitor, and regulate their voices.	Examine how vocal health can be positioned as part of undergraduate vocal learning rather than as supplementary advice.	RQ1, RQ3
Singers and music students face vocal health risks when vocal demands increase.	High school choir singers have shown poor voice hygiene and low voice-health knowledge (Yuksel et al., 2025). Preservice music teachers reported vocal fatigue, increased vocal effort, and changes in vocal health during student teaching, partly linked to overuse and increased vocal demands (Brunkan, 2018).	Vocal health risks are not limited to clinical populations; they may emerge in educational settings when students’ vocal demands increase but preventive knowledge and habits remain underdeveloped.	Identify what vocal health capacities undergraduate vocal students require for sustainable practice and performance.	RQ2, RQ3
Vocal health involves behavior and self-regulation, not only knowledge.	Music performance students reported weaker health responsibility, self-efficacy, and self-regulation than non-music health students (Ginsborg et al., 2009). Daily vocal health tracking has been shown to support monitoring of vocal hygiene and home-based voice therapy compliance (Sezin et al., 2025).	Vocal health education cannot be reduced to giving information; students need to translate knowledge into daily monitoring, decision-making, rest, hydration, and help-seeking behavior.	Understand vocal health as a self-regulatory learning process involving awareness, self-monitoring, behavioral regulation, and preventive action.	RQ2, RQ3
Vocal health education may improve awareness and self-management, but evidence remains context specific.	Online vocal health education improved self-perceived knowledge, confidence, awareness, and reported vocal health practices among music therapists (Liddell et al., 2026). Preventive voice training improved vocal function, vocal self-concept, and wellbeing among university personnel (Lücking and Claßen, 2026). Flow phonation exercises showed preliminary pedagogical value for undergraduate singers in training (Diaz et al., 2026).	Existing interventions show promise, but they do not directly establish what undergraduate vocal music students need before an intervention or learning resource is designed.	Conduct a needs analysis before proposing future vocal health learning support.	RQ2, RQ3
Vocal learning is psychologically demanding.	Self-regulated learning among conservatory students is shaped by teacher support, practice hours, performance anxiety, helplessness, and perceived independence (Zarza-Alzugaray et al., 2025). Vocal learning is also affected by positive emotion, psychological intervention, coping strategies, resilience, and mental health (He, 2020; Vasylevska-Skupa et al., 2024; Zhang, 2022).	Vocal health cannot be treated only as a physiological or technical issue because anxiety, confidence, motivation, and emotional readiness influence vocal use and performance.	Include psychological and emotional regulation within the concept of vocal health self-regulation.	RQ2, RQ3
The Chinese context shows limited pedagogical integration of vocal health.	A scoping review of vocal health literature in China found that vocal health remains insufficiently addressed in curricula and that studies are concentrated largely in medical fields rather than pedagogical settings (Guo and Mazlan, 2024).	There is insufficient evidence on how vocal health is understood as a pedagogical and psychological learning need in Chinese undergraduate vocal education.	Use ten selected vocal music textbooks and expert interviews to identify contextual issues, target needs, and learning needs.	RQ1, RQ2, RQ3
Needs analysis is required before instructional development.	Need analysis can clarify necessities, lacks, wants, target needs, and learning needs (Bocanegra-Valle, 2016; Pu et al., 2025). It is also useful for designing learning materials that are responsive to learners and stakeholders (Anwar et al., 2023; Mat et al., 2025).	Without need analysis, future vocal health materials risk being content-driven rather than evidence-informed, learner-responsive, and pedagogically grounded.	Establish an evidence-based foundation for future instructional support for vocal health self-regulation.	RQ3

Accordingly, this study conducts a qualitative needs analysis of vocal health self-regulation in undergraduate vocal music education. It examines ten selected vocal music textbooks, together with expert perceptions of students’ psychological, behavioral, pedagogical, and health-related learning needs. By focusing on target needs and learning needs, the study aims to identify the foundations required for developing more systematic support for vocal health awareness, self-monitoring, behavioral regulation, emotional readiness, and sustainable vocal practice. The contribution of the study is therefore needs-based and conceptual rather than psychometric or intervention-based. It does not claim to measure students’ psychological processes directly. Instead, it identifies expert-informed psychological learning needs that may guide future empirical research and instructional development in vocal health education.

Guided by this aim, the study addresses the following research questions:

1. How is vocal health represented in selected undergraduate vocal music textbooks in China?
2. What expert-informed psychological, behavioral, and pedagogical learning needs related to vocal health are identified by vocal education experts?
3. What target needs and learning needs should inform future support for vocal health self-regulation among undergraduate vocal music students?

## 2 Materials and methods

### 2.1 Research design

This study employed a qualitative needs-analysis design to examine vocal health self-regulation in undergraduate vocal music education. A qualitative approach was appropriate because the study sought to understand contextual issues, expert perceptions, professional judgment, and pedagogical needs rather than to test causal relationships or produce statistical generalization. Qualitative inquiry is suitable for exploring human experiences, attitudes, behaviors, and meanings within specific sociocultural and educational contexts (Cissé and Rasmussen, 2022). In this study, the needs-analysis phase was used to identify psychological, pedagogical, and health-related requirements for supporting undergraduate vocal students’ vocal health awareness, self-monitoring, behavioral regulation, emotional readiness, and sustainable vocal practice. The needs-analysis design was appropriate because needs analysis is commonly used as an early stage in educational and instructional development. It helps identify gaps between existing conditions and desired learning outcomes, thereby informing later curriculum, syllabus, or instructional material design (Gould et al., 2004; Dehnad et al., 2010; Mat et al., 2025). Although this article reports only on the needs-analysis phase, the broader doctoral study was informed by a design-oriented view of music pedagogy. In music education, design thinking has been argued to function as a pedagogical orientation that supports creative curriculum innovation, iterative problem-framing, and learner-responsive instructional development (Guo and Mazlan, 2025). The later phases of the broader study, including prototype development, expert validation, and pedagogical usability feedback, are outside the scope of this article.

### 2.2 Research context and conceptual orientation

The study was situated in undergraduate vocal music education in China. Undergraduate vocal students are expected to develop vocal technique, breath control, repertoire competence, musical interpretation, and performance readiness. However, vocal learning also requires students to manage vocal fatigue, practice intensity, rest, emotional tension, performance pressure, and preventive vocal care. These demands make vocal health relevant not only to vocal pedagogy, but also to educational psychology, health behavior, and self-regulated learning. In this study, vocal health self-regulation refers to students’ capacity to understand vocal health principles, monitor vocal condition, regulate practice intensity, apply preventive vocal care, manage emotional tension, and respond appropriately to signs of vocal discomfort. This conceptual orientation was informed by self-regulated learning theory, which views learners as active agents who monitor, evaluate, and regulate their own learning processes (Zimmerman, 2015), and by music education research that emphasizes autonomy, reflection, strategic practice, and performance development (McPherson et al., 2017). It was also informed by voice-related research that highlights the importance of daily vocal health tracking, self-report, motivation, compliance, and behavioral management in vocal health practice (Sezin et al., 2025).

### 2.3 Data sources

The study drew on two primary qualitative data sources: document mapping of ten selected vocal music textbooks and semi-structured interviews with five vocal music education experts. Relevant literature was used to frame the study conceptually and contextually, but the empirical data sources reported in the methodology were the selected textbooks and expert interview data. The textbook mapping examined how vocal music pedagogy, vocal technique, singing physiology, performance, psychological aspects of singing, and vocal health were represented in instructional materials. The expert interviews provided practice-based professional perspectives on undergraduate vocal pedagogy, students’ vocal health problems, current limitations in teaching materials, and the forms of support needed to develop vocal health self-regulation.

### 2.4 Document mapping of selected vocal music textbooks

A structured document mapping was conducted to contextualize how vocal health is represented in selected vocal music textbooks used in Chinese higher education. The mapping focused on ten Chinese-language vocal music textbooks that were pedagogically relevant to undergraduate vocal music education. The textbooks were purposively selected rather than statistically sampled. Selection was guided by their relevance to higher vocal music education, their focus on vocal pedagogy, vocal technique, singing physiology, repertoire learning, performance, or vocal teaching development, and their accessibility for chapter-level analysis. The purpose of this textbook mapping was therefore not to claim national textbook representativeness, but to provide contextual insight into how vocal health was positioned within a selected set of pedagogically relevant vocal music materials.

The textbook mapping was treated as contextual document analysis rather than a systematic review or bibliometric analysis. Document analysis is useful in qualitative research because documents can provide contextual, historical, institutional, and conceptual evidence relevant to the phenomenon under investigation (Bowen, 2009; Morgan, 2022). The mapping followed the logic of identifying, extracting, analyzing, and distilling relevant findings from selected materials, which is consistent with systematic approaches to qualitative document analysis (Dalglish et al., 2020).

To support systematic comparison, the ten textbooks were coded as B1 to B10 and examined at chapter level using a document analysis matrix. The matrix recorded bibliographic information, dominant instructional content, and thematic coverage across selected domains, including vocal technique, breath control, singing physiology, repertoire interpretation, pedagogy and teaching methods, musical literacy, psychological aspects of singing, performance, technology or innovation in teaching, and vocal health or vocal protection. The original Chinese titles, English translations, publication years, and authors of the selected textbooks are provided in Table 2.

**TABLE 2:** Selected vocal music textbooks and vocal-health coverage

Code	English title	Year / Author	Primary instructional orientation	Vocal-health / vocal-protection coverage
B1	*Jin Tielin’s Vocal Music Teaching Method*	Jin, 2013	Vocal teaching method; Chinese vocal art; staged vocal learning	Not directly addressed
B2	*Vocal Music Pedagogy*	Yang, 2007	Vocal pedagogy; teaching principles; teaching process; evaluation	Not systematically addressed
B3	*Research on Innovative Paths and Development Trends of Vocal Music Teaching in Higher Education*	Liu, 2022	Higher-education vocal teaching innovation; teaching models; teacher qualities	Not directly addressed
B4	*Vocal Music Pedagogy*	Cao, 2010	Vocal technique; vocal organs; vocal exercises; singing practice	Indirectly related through vocal organs and singing practice, but not systematically developed as vocal health
B5	*New Research on the Art of Vocal Music Teaching*	Zha, 2017	Vocal teaching art; teaching strategies; technique; performance practice	Not directly addressed
B6	*Research on Vocal Music Teaching and Dance Art*	Wang et al., 2019	Vocal teaching; singing physiology and psychology; stage practice; multimedia teaching	Indirectly related through physiology and psychology, but not systematically developed as vocal health
B7	*Research on Vocal Music Teaching Theory and Core Skills Training*	Zhang, 2021	Vocal theory; core skills; voice production; resonance; repertoire analysis	Not systematically addressed
B8	*Essays on Vocal Music Art Teaching*	Gao, 2022	Vocal art teaching; student learning methods; performance cases	Not directly addressed
B9	*Vocal Music Pedagogy for Higher Education*	Zhang, 2004	Higher-education vocal pedagogy; posture; breath; resonance; common singing problems	Briefly related through physiology and common singing problems, but fragmented
B10	*Vocal Tutorial*	Xu and Cui, 2013	Vocal tutorial; singing physiology; posture; breath; resonance; exercises	Briefly related through physiology and voice production, but fragmented

To respond to the need for transparent document classification, the selected textbooks were also classified according to their primary instructional orientation. The classification distinguished between vocal teaching method texts, vocal pedagogy texts, higher-education vocal teaching texts, vocal theory and skills texts, and vocal tutorial materials. Each textbook was then examined at chapter level to identify whether vocal-health or vocal-protection content was directly addressed, indirectly embedded through related topics such as vocal organs, singing physiology, voice production, or common singing problems, briefly or fragmentarily mentioned, or absent. This classification was used to clarify the pedagogical character of the selected materials and to make the basis for the document-mapping interpretation more transparent. A classified summary of the selected textbooks is presented in Table 2 below.

As shown in Table 2, the selected textbooks collectively cover vocal technique, pedagogy, performance, repertoire, singing physiology, and related learning issues. However, vocal health or vocal protection is either absent, indirectly embedded, briefly mentioned, or fragmented. No textbook in the selected sample presents vocal health as a systematic instructional domain involving self-monitoring, practice regulation, preventive behavior, rest management, and help-seeking. Overall, the document mapping provided the contextual basis for identifying the underdevelopment of vocal health in the selected instructional materials, while the expert interviews enabled these documentary patterns to be interpreted through practice-based professional perspectives.

### 2.5 Expert interview participants

Five vocal music education experts participated in semi-structured interviews. The participants were selected purposively because they had substantial experience in higher vocal music education, active involvement in vocal teaching, familiarity with vocal music teaching materials, and the ability to provide reflective professional perspectives on vocal pedagogy and vocal health. Purposive sampling was appropriate because the study required information-rich participants with domain-specific knowledge rather than statistically representative participants. In this sense, participant selection was guided by the relevance, reliability, and competence of informants in relation to the cultural and pedagogical domain under investigation (Tongco, 2007). This approach also strengthened the methodological fit between the research aim, participant expertise, and trustworthiness of the qualitative evidence, since purposive sampling is most defensible when the selected participants are closely matched to the objectives of the study (Campbell et al., 2020).

Expert interviews were suitable for this study because the research required access not only to explicit factual information about vocal music teaching, but also to situated professional judgement, pedagogical reasoning, and implicit knowledge developed through teaching practice. Döringer (2021) argues that expert interviews are particularly useful for investigating interpretive expert knowledge and the inner logic of decision-making within a field of action. Similarly, von Soest (2023) emphasizes that expert interviews can strengthen qualitative evidence when researchers clearly justify expert selection, address potential bias, and systematically capture expert knowledge. In the present study, the experts were therefore not treated as statistically representative respondents, but as knowledgeable informants whose professional perspectives could illuminate how vocal health is understood, represented, and needed within undergraduate vocal music education.

All five participants were experienced vocal educators working within the broad context of higher vocal music education in China. Their expertise was relevant to undergraduate vocal teaching, student vocal development, vocal pedagogy, and the use or evaluation of vocal music teaching materials. However, the interview protocol did not systematically collect detailed biographical data on each expert’s vocal tradition, genre specialization, voice type, or institutional curriculum context. Therefore, the study does not compare vocal health needs across different vocal genres or institutional models. Instead, the expert interviews are interpreted as professional perspectives from experienced higher vocal music educators within the studied Chinese undergraduate vocal education context. To protect participant confidentiality, the experts are identified in this article as Expert 1, Expert 2, Expert 3, Expert 4, and Expert 5.

### 2.6 Interview protocol

A semi-structured interview protocol was used to guide the expert interviews. Semi-structured interviews were appropriate because they provided a consistent structure across participants while allowing flexibility for experts to elaborate on their professional experiences, interpretations, and judgements. This balance between structure and openness is central to semi-structured interviewing, where a flexible protocol supports comparability across participants while allowing follow-up questions, probes, and situated meaning-making (Brinkmann and Kvale, 2015; DeJonckheere and Vaughn, 2019). The development of the protocol was also informed by methodological guidance on interview-guide construction, which emphasizes the need to clarify the interview purpose, draw on prior knowledge, formulate open-ended questions, and align prompts with the research aims (Kallio et al., 2016).

The protocol covered experts’ views on essential content for vocal music education, missing topics in existing method books, awareness and importance of vocal health, specific vocal health topics needed in instructional materials, student vocal health problems, causes of vocal issues, students’ vocal self-monitoring and practice regulation, and expectations for future integrated vocal music and vocal health learning resources. These domains were aligned with the study’s needs-analysis focus because they enabled the interviews to elicit expert perspectives on current pedagogical practice, students’ vocal health difficulties, and the forms of instructional support needed to develop vocal health self-regulation. The core semi-structured interview prompts relevant to the present article are provided in Table 3 to support transparency and transferability.

**TABLE 3:** Core semi-structured interview prompts used for the needs analysis

Interview focus	Core prompt	Follow-up prompts
Essential content for vocal music education	Can you list the key topics or sections you believe are essential for a comprehensive vocal music education method book?	Which areas are most important for undergraduate vocal students? Why?
Missing content in existing materials	What specific topics or issues do you think are frequently missing from current vocal music education method books?	Can you give examples based on your teaching experience?
Vocal health awareness	Are you familiar with vocal health?	How do you understand vocal health in relation to vocal music learning?
Importance of vocal health	Do you think vocal health-related content is important for a vocal music education method book?	Why is vocal health important for undergraduate vocal students?
Specific vocal health content	What specific vocal health topics should be included in a vocal music education method book?	Can you explain why these topics are important? Can you give an example?
Student vocal problems	Based on your teaching experience, what vocal health problems do undergraduate vocal students commonly experience?	What usually causes these problems?
Self-regulation and practice behavior	How do students usually monitor, protect, or regulate their voices during practice?	What support do students need to improve self-monitoring, practice regulation, rest, and vocal care?
Integrated instructional support	How do you envision a method book or learning resource that integrates both vocal music education and vocal health?	What key features should it have?
Format and organization	What format, layout, or organizational strategies would make such a resource useful for instructors and students?	Should it include visuals, exercises, reflective tasks, or practice guidance?
Potential challenges	What obstacles might arise in developing or using a vocal music education resource that includes vocal health?	How could these challenges be addressed?

The full doctoral interview protocol also included questions related to later design, validation, and usability phases of the broader study. However, the present article reports only the qualitative needs-analysis phase. Therefore, this supplementary table presents the core prompts most directly relevant to the article’s focus on textbook representation, expert-identified vocal health needs, and future support for vocal health self-regulation.

Although several prompts allowed experts to discuss confidence, motivation, emotional expression, anxiety, and performance readiness, the interview schedule did not systematically examine teacher–student relationship quality, studio power relations, emotional safety, or one-to-one pedagogical dynamics as independent areas of inquiry. These relational aspects are therefore treated as outside the central scope of the present article and are acknowledged as an area for future research.

### 2.7 Data collection procedure

The five interviews were conducted online between 1 August 2024 and 5 August 2024. Based on the informed consent documents, each participant was asked to participate in an interview lasting approximately 40 min. Before each interview, participants were informed about the purpose of the study, the voluntary nature of participation, confidentiality, the right to withdraw, permission for audio recording, and the intended use of anonymized data for publication and presentation. These procedures were consistent with qualitative interview practice, where careful planning, consent, privacy, confidentiality, and systematic recording are important for ethical and credible interview data collection (Bolderston, 2012). At the beginning of each interview, the researcher confirmed informed consent, voluntary participation, permission for audio recording, and the participants’ understanding of confidentiality.

The interviews followed the semi-structured protocol described in Section “2.6 Interview protocol”, while allowing follow-up questions and probes when participants introduced relevant professional experiences or examples. This flexibility enabled the researcher to obtain comparable information across experts while also capturing context-specific insights into vocal teaching practice, student vocal health problems, and perceived gaps in existing instructional materials. The interviews explored experts’ perceptions of vocal music education and vocal health. The transcript data showed that experts discussed recurring teaching emphases such as vocal technique, breath control, pitch training, song interpretation, stage performance, and emotional expression. They also discussed student vocal problems such as vocal fatigue, hoarseness, excessive vocal strain, insufficient rest, lack of self-protection awareness, anxiety, and limited systematic vocal health content in existing teaching materials. The interviews were conducted and transcribed in Chinese. Relevant participant quotations were subsequently translated into English for reporting, with attention given to preserving their contextual and conceptual meanings across languages (van Nes et al., 2010). The transcripts were reviewed for analytic use before coding and thematic interpretation.

### 2.8 Analytic framework

The analysis was guided by a needs-analysis framework focusing on target needs and learning needs. In this study, target needs referred to what undergraduate vocal students require in order to sustain healthy vocal learning and performance. Target needs were interpreted through necessities, lacks, and wants. Necessities referred to vocal health capacities are regarded as essential for continued vocal learning and performance development. Lacks referred to missing or insufficient elements in current teaching materials, student awareness, or pedagogical practice. Wants referred to the types of content, resources, and support that experts recommended for future vocal health instruction. Learning needs referred to the instructional processes, strategies, resources, and learning conditions required to help students develop vocal health self-regulation. These included support for vocal health awareness, self-monitoring, behavioral regulation, emotional readiness, rest management, sustainable practice, and help-seeking behavior. This framework was informed by needs-analysis literature that conceptualizes needs in terms of necessities, lacks, wants, goals, difficulties, preferences, expectations, and constraints (Bocanegra-Valle, 2016), and by music education needs-analysis research that demonstrates how target needs and learning needs can inform pedagogical development in higher education music contexts (Pu et al., 2025).

### 2.9 Data analysis

Data analysis proceeded in two stages: document mapping of selected vocal music textbooks and thematic analysis of expert interview data. The two stages were analyzed separately before being synthesized through the needs-analysis framework. This analytic sequence enabled the study to first identify how vocal health was represented in selected instructional materials and then interpret these documentary patterns through expert professional perspectives. First, the document mapping examined the ten selected vocal music textbooks at chapter level. Each textbook was coded as B1 to B10 and analyzed using the document analysis matrix described in Section “2.4 Document mapping of selected vocal music textbooks”. The analysis recorded dominant instructional domains, including vocal technique, breath control, singing physiology, repertoire interpretation, pedagogy and teaching methods, musical literacy, psychological aspects of singing, performance, technology or innovation in teaching, and vocal health or vocal protection. The aim was not to conduct a page-level quantitative content analysis, but to identify the presence, absence, indirect embedding, or fragmented treatment of vocal-health-related content within the selected instructional materials. This procedure enabled the textbook findings to be interpreted systematically rather than impressionistically.

Second, the expert interview data were analyzed thematically. Thematic analysis was appropriate because it enables researchers to identify, organize, and interpret patterns of meaning across qualitative data (Braun and Clarke, 2006; Herzog et al., 2019; Mason and Francis, 2022). Thematic analysis is widely used in qualitative research and is suitable for analyzing interview data in a systematic and transparent manner (Cernasev and Axon, 2023; Nowell et al., 2017). The analysis began with repeated reading of the interview transcripts to develop familiarity with the data. Initial codes were generated from meaningful segments of text, including references to vocal technique, breath control, practice habits, vocal fatigue, vocal protection, psychological barriers, motivation, self-confidence, learning difficulties, and limitations of current teaching materials. Related codes were then grouped into broader themes and interpreted in relation to the study’s needs-analysis focus.

The analysis combined inductive and conceptually guided coding. It was inductive because codes and themes were generated from repeated patterns in the expert interview data rather than imposed in advance. At the same time, the interpretation was conceptually guided by the needs-analysis distinction between target needs and learning needs, including necessities, lacks, and wants. This hybrid logic was appropriate because the analysis needed to remain grounded in expert accounts while also being interpreted through a guiding needs-analysis framework (Fereday and Muir-Cochrane, 2006). Target needs were interpreted in relation to what undergraduate vocal students require, what is currently lacking, and what experts believe should be prioritized. Learning needs were interpreted as the pedagogical support, content, strategies, and learning conditions required to develop vocal health self-regulation. To strengthen analytic transparency, the themes and needs-analysis categories were checked against representative expert evidence. Recurring expert statements were organized into an evidence matrix showing how the categories of necessities, lacks, wants, and learning needs were derived. This process helped establish a traceable link between expert statements, initial codes, thematic interpretation, and needs-analysis categories. The evidence matrix is reported in Section “3.3 Target needs for vocal health self-regulation” as part of the presentation of target needs and learning needs.

### 2.10 Trustworthiness

Several strategies were used to strengthen trustworthiness. Trustworthiness in qualitative research is commonly associated with credibility, transferability, dependability, and confirmability (Lincoln and Guba, 1985; Shenton, 2004; Korstjens and Moser, 2018). In this study, credibility was supported through triangulation between document mapping and expert interview findings. The textbook mapping provided documentary evidence of how vocal health was represented in selected instructional materials, while the expert interviews provided practice-based professional perspectives on student vocal health needs, pedagogical gaps, and future instructional support. This helped reduce reliance on a single data source and enabled the findings to be interpreted through convergence between documentary and professional evidence.

Procedural consistency was supported through the use of a semi-structured interview protocol. The protocol ensured that all expert participants were asked about comparable domains, including essential vocal music education content, missing topics in existing materials, vocal health awareness, student vocal problems, and future integrated vocal health support. At the same time, the semi-structured format allowed flexibility for experts to elaborate on their professional experiences and provide contextual examples. The core prompts are provided in Table 3 to support transparency and transferability.

After transcription, the researcher shared the interview transcripts with the respective participants for confirmation of accuracy where possible. This process focused on transcript accuracy rather than full participant validation of the thematic interpretation. Therefore, the study does not claim comprehensive member checking of the findings. Dependability and confirmability were strengthened through repeated reading of transcripts, initial coding, grouping of related codes, thematic interpretation, and synthesis through the needs-analysis framework. To make the analytic process more traceable, representative expert statements were organized into an evidence matrix showing how the categories of necessities, lacks, wants, and learning needs were derived. This evidence matrix is reported in Section “3.3 Target needs for vocal health self-regulation” and Table 4.

**TABLE 4:** Representative expert evidence supporting the needs-analysis categories

Needs-analysis category	Representative expert evidence	Initial code	Interpretation
Necessities	E1: “Vocal health is the foundation of vocal music learning.” E4: “Without [vocal health], the learning process cannot continue.” E5: “There is nothing more important than this… vocal health is the most fundamental element.”	Vocal health as foundation; learning continuity; performance quality	Vocal health was coded as a necessity because experts described it as essential for learning, performance, and long-term vocal development.
Lacks	E1: Vocal health is “briefly [mentioned] in appendices or supplementary materials.” E2: “Content on vocal health is relatively scarce, often mentioned only in appendices and lacking systematic guidance.” E4: Existing textbooks provide only basic tips and lack systematic guidance.	Limited vocal health content; supplementary treatment; lack of systematic guidance	This was coded as a lack because experts identified insufficient structured vocal health content in current materials.
Lacks	E1: Vocal problems may result from incorrect technique, excessive vocal-cord exertion, intense practice without rest, and lack of self-care awareness. E4: “Excessive practice intensity or improper techniques are major causes of vocal issues.” E5: Students often neglect rest and may continue using the voice intensely.	Over-practice; poor practice regulation; lack of rest; weak self-protection awareness	This was coded as a lack because experts identified missing student capacities in self-monitoring, practice regulation, and preventive vocal care.
Wants	E2: Future materials should include vocal health concepts, protection methods, common vocal diseases, vocal relaxation exercises, and images of the vocal cords and larynx. E5: Materials should include vocal technique, breathing methods, prevention of common vocal disorders, daily voice protection, practice-schedule adjustment, and diagrams of vocal structures.	Desired content; disease prevention; visual aids; vocal care strategies	This was coded as wants because experts explicitly recommended what future vocal health materials should include.
Learning needs	E3: Vocal health affects students’ psychological state and motivation to learn. E4: Anxiety and nervousness may cause vocal tightness, dry throat, and unstable tone. E5: Students should pay attention to their vocal condition and provide timely feedback when discomfort occurs.	Psychological readiness; confidence; anxiety management; self-monitoring; emotional regulation	This was coded as a learning need because vocal health learning requires emotional regulation, self-monitoring, and performance-readiness support, not only technical instruction.

Transferability was supported by providing contextual information about the selected textbooks, the expert sampling logic, and the Chinese undergraduate vocal music education context in which the study was situated. To reduce overstatement, the findings are reported as evidence from selected textbook materials and expert perceptions rather than as generalisable claims about all undergraduate vocal music education in China

### 2.11 Ethical considerations

Ethical approval was obtained from the authors’ institutional Human Research Ethics Committee. The approval details have been withheld during peer review to preserve author anonymity and will be provided in the non-anonymized title page or final manuscript if required. All expert participants received a respondent information sheet and informed consent form before participation. The consent forms explained the study purpose, interview procedure, approximate interview duration, possible risks, confidentiality procedures, voluntary participation, right to withdraw, permission for audio recording, and the use of anonymized data for publication and presentation.

At the beginning of each interview, consent was reconfirmed verbally before data collection proceeded. Participant identities were anonymized in the reporting of findings, and no real names are used in the article. Interview data were treated confidentially and used only for the purposes described in the participant information sheet and informed consent form. Because the article reports expert perceptions and document mapping rather than student intervention data or clinical voice assessment, the study was considered minimal risk.

## 3 Results

This section presents the results of the qualitative needs analysis. The results are organized according to the three research questions. First, the section reports on how vocal health was represented in the selected vocal music textbooks. Second, it presents expert interview findings concerning undergraduate vocal students’ vocal health-related psychological, behavioral, and pedagogical needs. Third, it synthesizes the document mapping and expert interview findings into target needs and learning needs for supporting vocal health self-regulation in undergraduate vocal music education.

### 3.1 Representation of vocal health in selected vocal music textbooks

The document mapping identified limited and fragmented representation of vocal health in the selected vocal music textbooks. Across the ten textbooks, the dominant instructional areas were vocal technique, breath control, singing physiology, repertoire interpretation, pedagogy, teaching methods, performance, and psychological aspects of singing. These areas reflect the dominant instructional structure of undergraduate vocal music education, where technical and artistic development remain central.

However, vocal-health or vocal-protection content was not developed as a systematic instructional domain in the selected materials. In several textbooks, vocal health was indirectly embedded through related topics such as vocal organs, singing physiology, voice production, posture, breath, or common singing problems. In others, vocal health or vocal protection was absent or only briefly and fragmentarily mentioned. None of the selected textbooks presented vocal health as a structured learning domain involving self-monitoring, practice regulation, preventive behavior, rest management, emotional readiness, or help-seeking. This finding suggests that vocal health may be implicitly connected to technique, physiology, or performance preparation, but it is not explicitly organized as a self-regulatory learning process. The textbook mapping therefore provided an important contextual basis for the expert interviews, which examined how experienced vocal educators perceive students’ vocal health needs in practice.

### 3.2 Expert-identified vocal health needs among undergraduate vocal students

The expert interview findings were interpreted in relation to the semi-structured interview domains described in Section “2.6 Interview protocol” and Table 3. The themes reported below were derived from recurring patterns across the five expert accounts and were interpreted through the needs-analysis focus of the study. In particular, the psychological findings emerged from expert comments on confidence, anxiety, motivation, emotional expression, and performance readiness. They should not be interpreted as a systematic analysis of teacher–student relationship quality, studio power relations, or one-to-one pedagogical dynamics, which were outside the central scope of the present article.

The expert interviews generated five major themes: technique-centered vocal training, vocal health as an underdeveloped learning domain, student vocal problems as behavioral and self-regulatory issues, psychological dimensions of vocal health, and the need for systematic and practical vocal health learning support. These themes were constructed by comparing repeated patterns across the five expert accounts and interpreting them in relation to the needs-analysis framework.

#### 3.2.1 Technique-centered vocal training remains dominant

All five experts described vocal music teaching as strongly centered on vocal technique, breath control, pitch training, song interpretation, and stage performance. These areas were viewed as foundational to students’ vocal development. Expert 1 explained that vocal music teaching usually covers “singing techniques,” “breath control,” “music theory,” and “performance techniques on stage.” Similarly, Expert 2 described vocal technique, breath control, pitch training, and repertoire performance as “fundamental elements of vocal music education.” Expert 4 also emphasized that correct vocal technique is foundational because it can help prevent vocal injury. This dominance of technique is not inherently problematic. The experts agreed that correct vocal technique and breath control are essential for producing sound safely and expressively. However, the findings suggest that a technique-centered model becomes insufficient when it is not accompanied by structured vocal health awareness. In other words, students may learn how to sing, but not necessarily how to monitor, protect, and sustain the voice that enables singing.

#### 3.2.2 Vocal health is treated as important but underdeveloped

A strong pattern across the interviews was that experts regarded vocal health as essential, while also acknowledging that it is insufficiently represented in existing teaching materials. Expert 1 stated that “vocal health is the foundation of vocal music learning” and explained that poor vocal health can disrupt practice, reduce learning efficiency, and affect students’ future vocal development. Expert 4 described vocal health as a condition for learning continuity, stating that “without it, the learning process cannot continue.” Expert 5 similarly positioned vocal health as the most fundamental element of vocal music learning, explaining that “there is nothing more important than this” because even slight hoarseness or serious vocal-cord damage can affect students’ learning and singing quality.

Despite this shared recognition, experts reported that vocal health content in current materials is limited, fragmented, or supplementary. Expert 1 stated that current vocal music teaching materials in China usually mention vocal health only briefly in appendices or supplementary materials and that most materials focus more on singing technique and repertoire training. Expert 2 similarly observed that “content on vocal health is relatively scarce, often mentioned only in appendices and lacking systematic guidance.” Expert 4 also stated that existing vocal music textbooks give limited attention to vocal health and usually provide basic voice-care tips rather than systematic guidance. This finding indicates a clear gap between expert understanding and instructional-material design. Experts recognize vocal health as foundational, but formal materials do not yet appear to support this recognition in a systematic way. For this reason, vocal health was interpreted as a necessary but underdeveloped learning domain.

#### 3.2.3 Student vocal problems reflect behavioral and self-regulatory gaps

Experts consistently reported that students experience vocal fatigue, hoarseness, strain, and other vocal problems during practice. These problems were attributed not only to technical errors, but also to behavioral and self-regulatory factors such as over-practice, insufficient rest, lack of warm-up, poor practice planning, limited self-protection awareness, and failure to recognize early signs of vocal strain. Expert 1 linked students’ vocal problems to incorrect singing techniques, excessive vocal-cord exertion, long periods of intense practice without adequate rest, and lack of self-care awareness. Expert 2 explained that students may experience vocal problems because of insufficient knowledge about proper voice use, incorrect practice methods, continuous practice without rest, and poor self-management.

Expert 4 attributed vocal problems to “excessive practice intensity or improper techniques,” adding that some students may not realize the harm of over-practising. Expert 5 also noted that students often neglect rest during practice and may continue using the voice intensely even when discomfort appears. This theme is important because it reframes vocal health as more than technical correction. Students need to develop the capacity to regulate practice intensity, monitor vocal condition, rest appropriately, and respond to discomfort. These are self-regulatory behaviors. Therefore, vocal health education should include practical guidance on how students can manage their own vocal learning, rather than only information about the voice.

#### 3.2.4 Vocal health is connected to psychological dimensions and performance readiness

Several experts linked vocal health with confidence, anxiety, motivation, stage performance, and emotional expression. Expert 3 stated that vocal health affects students’ psychological state and motivation to learn. The expert described a student who developed self-doubt because of vocal problems but regained confidence through vocal recovery and psychological support. This example shows that vocal problems can affect students’ self-perception, participation, and motivation. Expert 4 also noted that psychological factors can contribute to vocal issues.

The expert explained that anxiety and nervousness before performances or examinations may lead to vocal tension, dry throat, unstable tone, and other difficulties during singing. For this reason, Expert 4 emphasized psychological guidance, confidence-building, relaxation, and emotional management as part of vocal teaching. Expert 2 and Expert 5 also connected vocal learning with confidence, emotional expression, and students’ holistic development. This finding is central to the article’s psychological framing. From the experts’ perspectives, vocal health was not only a physical or physiological concern but was also perceived as connected to students’ emotional state, confidence, anxiety, and readiness to perform. Therefore, vocal health self-regulation should include emotional awareness and psychological regulation, not only vocal hygiene.

#### 3.2.5 Experts want practical, visual, and systematic vocal health support

When asked what should be included in future vocal health learning materials, experts recommended a more systematic and applied structure. Suggested content included vocal anatomy, vocal-cord structure, principles of sound production, vocal health concepts, daily vocal care, prevention of vocal disorders, correct vocal technique, practice-intensity adjustment, vocal relaxation exercises, hydration, diet, rest, and medical referral when necessary. Expert 1 recommended that future materials should include vocal music concepts, singing techniques, breath control, vocal health concepts, illustrations, vocal protection methods, daily care, and disease prevention. Expert 2 recommended content on vocal techniques, vocal health maintenance, common vocal diseases, vocal relaxation exercises, and images of the vocal cords and larynx.

Expert 3 suggested that materials should include vocal-cord physiology, prevention of common vocal issues, maintenance strategies, practical examples, and illustrations of singing-related muscles. Expert 4 recommended scientific vocal methods, breath training, daily vocal care, reasonable practice planning, prevention and treatment of vocal disorders, and anatomical illustrations. Expert 5 similarly recommended vocal technique concepts, breathing methods, prevention of common vocal disorders, daily voice protection, practice-schedule adjustment, and diagrams of vocal structures. This theme shows that experts do not merely want more information. They want usable learning support: visual, practical, accessible, and directly connected to students’ daily vocal practice. This supports the interpretation that future vocal health education should move beyond general advice and provide structured pedagogical support for self-regulation.

### 3.3 Target needs for vocal health self-regulation

Based on the document mapping and expert interviews, the target needs were interpreted through three categories: necessities, lacks, and wants. In this study, necessities refer to vocal health capacities that experts consider essential for students continued vocal learning and performance development. Lacks refer to missing or insufficient elements in current teaching materials, student awareness, or pedagogical practice. Wants refer to the types of content, resources, and support that experts recommended for future vocal health instruction. To make the analytic process transparent, representative expert interview data were organized into an evidence matrix, as presented in Table 4. The categories of necessities, lacks, wants, and learning needs were derived from recurring expert statements and interpreted through the needs-analysis framework. A statement was coded as a necessity when experts described vocal health as foundational, essential, or required for continued vocal learning. A statement was coded as a lack when experts identified missing or insufficient content, awareness, behavior, or support. A statement was coded as a want when experts recommended what future teaching materials or instruction should include. A statement was coded as a learning need when experts described the pedagogical processes, strategies, or learning conditions required to develop students’ vocal health self-regulation.

This evidence matrix indicates that the needs-analysis categories were not imposed only from theory but were grounded in repeated expert evidence and interpreted through the needs-analysis framework. The category of necessities was derived from experts’ repeated positioning of vocal health as foundational to learning continuity, performance quality, and future vocal development. The category of lacks was derived from two forms of absence: insufficient vocal health content in existing materials and students’ limited self-protection awareness. The category of wants was derived from experts’ recommendations for future teaching materials, including vocal anatomy, vocal care, disease prevention, visual illustrations, and practical exercises. Finally, the category of learning needs was derived from experts’ descriptions of the processes required to develop vocal health self-regulation, including self-monitoring, practice regulation, rest, hydration, relaxation exercises, emotional regulation, and help-seeking.

### 3.4 Learning needs for supporting vocal health self-regulation

The findings indicate several learning needs required to support vocal health self-regulation. First, students need integrated instruction linking technique and vocal health. Experts did not separate vocal technique from health. Instead, they repeatedly connected incorrect technique, excessive strain, and poor practice habits with vocal fatigue and hoarseness. Therefore, vocal techniques should be taught as health-informed practice rather than as isolated sound-production skills. Second, students need self-monitoring routines. Expert accounts suggested that students may fail to recognize or respond appropriately to vocal discomfort. Expert 5 specifically emphasized that students should pay attention to their vocal condition during practice and provide timely feedback when discomfort occurs. Learning materials should therefore guide students to observe vocal fatigue, hoarseness, throat discomfort, tension, and recovery needs.

Third, students need behavioral regulation strategies. These include managing practice duration, warming up before practice, taking rest, maintaining hydration, avoiding excessive voice use, and making appropriate lifestyle adjustments. The experts’ repeated emphasis on rest, hydration, practice adjustment, and avoidance of overuse indicates that vocal health learning should include daily behavioral regulation rather than general advice alone. Fourth, students need psychological and emotional support related to vocal learning and performance readiness. Expert accounts link anxiety, low confidence, performance pressure, and emotional tension with vocal production and vocal health.

Therefore, vocal health education should include reflective prompts, relaxation strategies, confidence-building, and emotional readiness for performance. However, these findings should be interpreted as expert-identified psychological learning needs rather than as direct evidence from student psychological assessment. Fifth, students need practical and visual learning resources. Experts recommended anatomical illustrations, applied exercises, vocal relaxation techniques, and concrete daily-care strategies. These resources can help students understand the voice not only conceptually, but also practically and experientially.

### 3.5 Summary of the needs analysis

The needs analysis shows that, within the selected textbook materials and expert accounts examined in this study, undergraduate vocal music education would benefit from a shift from a primarily technique-centered model toward a health-integrated and self-regulatory model of vocal learning. This interpretation was derived from the convergence between the document mapping and expert interview evidence. The textbook mapping identified limited and fragmented representation of vocal health in the selected instructional materials. The expert interviews then clarified how these gaps are perceived in teaching practice, particularly through expert-reported concerns about students’ vocal fatigue, hoarseness, poor practice regulation, limited self-protection awareness, anxiety, and the need for more systematic vocal health support.

The cross-data synthesis is summarized in Table 5, which links each synthesized finding to its evidence basis and implication for vocal health self-regulation. This table was included to make the interpretive movement from textbook mapping and expert interview evidence to learning-need implications more transparent.

**TABLE 5:** Summary of needs analysis findings and implications for vocal health self-regulation

Synthesized finding	Evidence basis	Implication for vocal health self-regulation
Vocal education remains strongly centered on technique, breath control, pitch, repertoire, and performance.	The textbook mapping showed that the selected textbooks mainly emphasized vocal technique, breath control, singing physiology, repertoire interpretation, pedagogy, teaching methods, performance, and psychological aspects of singing. Experts also described technique, breath control, pitch training, repertoire interpretation, and stage performance as frequently taught content.	Vocal health should be integrated into technical instruction rather than treated as a separate or supplementary topic.
Vocal health is recognized as essential but remains underdeveloped in the selected materials and expert accounts.	Across the ten selected textbooks, only a small number included content indirectly or briefly related to vocal health, such as vocal organs, singing physiology, common singing problems, or voice production. However, none developed vocal health as a systematic instructional domain. Experts also stated that vocal health content in current materials is limited, fragmented, or placed only in appendices or supplementary sections.	Vocal health should be developed as a structured learning domain within undergraduate vocal music education, particularly in relation to the selected materials and expert-identified needs.
Expert-reported student vocal problems are linked to behavioral and self-regulatory gaps.	Experts attributed vocal fatigue, hoarseness, strain, and misuse to incorrect technique, excessive practice, insufficient rest, poor warm-up habits, lack of self-protection awareness, and weak practice planning.	Students need explicit support for self-monitoring, practice regulation, rest, vocal care, and early recognition of vocal discomfort.
Vocal health is connected to psychological dimensions and performance readiness.	Experts linked vocal problems with anxiety, low confidence, nervousness, emotional tension, motivation loss, and reluctance to perform.	Vocal health self-regulation should include emotional regulation, confidence-building, relaxation, and performance-readiness strategies.
Experts call for practical, visual, and systematic vocal health resources.	Experts recommended content on vocal anatomy, vocal-cord structure, vocal care, disease prevention, correct vocal technique, practice-intensity adjustment, relaxation exercises, illustrations, and daily protection strategies.	Future instructional support should include practical exercises, visual aids, reflective activities, and applied self-care routines.

In response to RQ1, the document mapping identified how vocal health was represented in the selected undergraduate vocal music textbooks. The findings showed that the selected textbooks mainly emphasized vocal technique, breath control, singing physiology, repertoire interpretation, pedagogy, teaching methods, performance, and psychological aspects of singing. Vocal health or vocal protection was either absent, indirectly embedded, briefly mentioned, or fragmented, and was not developed as a systematic instructional domain.

In response to RQ2, the expert interviews showed that students’ vocal health needs were perceived as technical, behavioral, psychological, and pedagogical. Experts consistently connected vocal health problems with practice habits, self-protection awareness, emotional state, confidence, motivation, and access to systematic guidance. These findings suggest that vocal health support should address not only what students know about the voice, but also how they monitor, regulate, and protect the voice during practice and performance preparation.

In response to RQ3, the study identifies vocal health self-regulation as a key target learning need. The findings indicate that students require structured support for vocal health awareness, self-monitoring, behavioral regulation, emotional readiness, sustainable practice, and help-seeking. The categories of necessities, lacks, wants, and learning needs were therefore derived from the evidence: vocal health was identified as necessary for learning continuity; current materials and expert-reported student behaviors showed important lacks; experts wanted systematic and practical resources; and students were perceived to need pedagogical support to develop self-regulated vocal health practices.

Overall, the results support the central argument of this article: vocal health should not be treated merely as physiological knowledge or supplementary vocal hygiene advice. Instead, within the studied context, it should be understood as a self-regulated learning process through which undergraduate vocal students learn to understand, monitor, protect, and sustain their voices throughout musical development.

## 4 Discussion

This study conducted a qualitative needs analysis to examine vocal health self-regulation in undergraduate vocal music education. By combining document mapping of ten selected vocal music textbooks with semi-structured interviews with five vocal music education experts, the study identified a discrepancy between the recognized importance of vocal health and its limited systematic representation in the selected instructional materials and expert accounts. Experts consistently positioned vocal health as foundational to learning continuity, performance quality, and long-term vocal development. However, the document mapping showed that vocal-health or vocal-protection content was absent, indirectly embedded, briefly mentioned, or fragmented in the selected textbooks, rather than developed as a systematic instructional domain.

The central contribution of this study is not a national curriculum audit, a psychometric investigation, or an evaluation of intervention effectiveness. Rather, the study contributes to a needs-based conceptual reframing of vocal health as an expert-informed psychological and pedagogical learning need involving awareness, self-monitoring, behavioral regulation, emotional readiness, sustainable practice, and help-seeking. This reframing is important because vocal health is often treated as physiological knowledge, supplementary hygiene advice, or teacher-provided correction. The present findings suggest that future vocal health education should also support students’ capacity to monitor, interpret, and regulate their own vocal behaviors. However, because the study did not directly measure students’ anxiety, motivation, self-efficacy, emotional regulation, or self-regulated learning, the psychological contribution should be understood as identifying relevant learning needs rather than demonstrating psychological mechanisms.

### 4.1 Reframing vocal health as a self-regulated learning need

The results support the argument that vocal health should be understood as a self-regulated learning process rather than simply as physiological knowledge, vocal hygiene advice, or teacher-corrected technique. Expert accounts repeatedly described students’ vocal problems as associated with incorrect technique, excessive practice, insufficient rest, weak self-protection awareness, poor warm-up habits, and limited capacity to recognize early signs of vocal strain. These results suggest that vocal health depends not only on what students know, but also on how they monitor, judge, and regulate their behavior during daily practice.

This interpretation aligns with self-regulated learning theory, which views learners as active agents who set goals, monitor performance, regulate strategies, and reflect on outcomes (Zimmerman, 2015). In music education, self-regulated learning has been associated with effective practice, performance preparation, autonomy, and reflective musicianship (McPherson et al., 2017). Recent music psychology research further supports this interpretation. López-Íñiguez and McPherson (2020) showed that self-regulated learning and self-determination theory can be used to understand how musicians regulate motivation, cognition, behavior, affect, and performance preparation over time. Similarly, dos Santos Silva et al. (2024) found that self-regulated learning in music practice involves practice organization, personal resources, and external resources, with more experienced musicians showing stronger metacognitive engagement. These studies support the present argument that vocal students need to regulate not only musical practice, but also vocal condition, fatigue, rest, emotional tension, and help-seeking behavior.

This is a critical shift. In much vocal pedagogy, vocal health is easily reduced to advice about hydration, rest, warm-up, or avoiding vocal misuse. While such advice is important, it is insufficient if students are not supported to apply it consistently within their own practice routines. The results therefore suggest that vocal health education should move from information provision to self-regulatory capacity building. This framing is consistent with Sezin et al. (2025), whose work on daily vocal health tracking highlights the importance of self-report, compliance, motivation, and behavioral management in vocal health practice. It also aligns with dos Santos Silva et al. (2025), who argued that feedback directed at the self-regulation level can help music students define goals, solve practice problems, evaluate strategies, adapt behavior, monitor their own practice, and assess their performance. However, unlike clinical or therapy-oriented contexts, undergraduate vocal education requires these capacities to be developed preventively, before serious vocal problems occur.

### 4.2 The pedagogical gap: technique is central, but health is peripheral

The results suggest that, within the selected textbook materials and expert accounts, undergraduate vocal music education remains strongly centered on vocal technique, breath control, pitch, repertoire, interpretation, and performance. These areas are necessary and were affirmed by the experts as core components of vocal learning. The issue is not that technique-centered education is irrelevant, but that technique becomes pedagogically incomplete when it is not accompanied by systematic vocal health self-regulation. The textbook mapping strengthens this point. The selected textbooks largely emphasized technique, singing physiology, repertoire, pedagogy, performance, and psychological aspects of singing. However, vocal-health or vocal-protection content was either absent, indirectly embedded through related topics, briefly mentioned, or fragmented. None of the selected textbooks developed vocal health as a systematic instructional domain involving self-monitoring, practice regulation, preventive behavior, rest management, and help-seeking. This suggests that vocal health may be acknowledged indirectly through discussions of technique, physiology, posture, breath, voice production, or common singing problems, but it is not yet constructed as a structured learning domain in the selected materials. This is important because students may learn how to produce sound but not how to monitor vocal load, identify harmful practice patterns, manage recovery, or respond to early signs of strain.

This result is consistent with Flynn’s (2020) observation that vocal health education in undergraduate performing arts programmes is often inconsistent, and that students may receive general vocal hygiene information without systematic instruction on vocal injury, voice disorders, and long-term management. Lau et al. (2026) similarly found that conservatorium singing students’ limited knowledge of vocal anatomy and physiology may constrain their engagement with vocal hygiene and help-seeking. The present study also aligns with wider music psychology research showing that music students’ health-related knowledge, attitudes, and behaviors require explicit pedagogical attention. Araújo et al. (2017), for example, showed that higher education music students’ health behaviors, stress management, and health responsibility are important concerns within music training. Rosset et al. (2022) further demonstrated that music students may enter higher education with substantial practice demands and that courses on musicians’ health can increase students’ knowledge and skills concerning health risks and protective measures. These studies support the present finding that health-related learning should not be postponed or treated as incidental but embedded into the structure of music education.

The pedagogical issue is therefore not only about whether vocal health content exists, but how it is organized within learning. If vocal health appears only as brief advice, appendices, or supplementary information, students may not learn how to integrate vocal care into practice decisions, technique development, and performance preparation. This is particularly important because recent Frontiers in Psychology research on vocal music training suggests that feedback and reflection can support metacognition and singing performance (Li et al., 2025). Such evidence reinforces the need to move beyond teacher correction and technical repetition toward learning processes that cultivate monitoring, reflection, and self-regulatory judgement. Therefore, moving beyond technique-centered vocal education does not mean reducing the importance of technique. Rather, it means teaching vocal technique as health-informed practice. Breath control, resonance, repertoire preparation, warm-up, diction, projection, and performance preparation should be connected to vocal care, self-monitoring, practice planning, rest, and recovery. This integration would allow students to understand vocal health not as an external add-on, but as part of competent singing. In this sense, the present study supports Lubert et al.’s (2025) call for a culture of care in music higher education, where health and wellbeing are positioned as collective and institutional responsibilities rather than individual afterthoughts.

### 4.3 Vocal health as behavioral and preventive practice

The results also indicate that expert-reported vocal health problems are partly behavioral and preventive in nature. Experts linked vocal fatigue, hoarseness, strain, and misuse to over-practice, inadequate rest, weak self-care awareness, poor practice planning, and delayed response to discomfort. These results are consistent with broader musicians’ health research, which emphasizes behavior change, injury prevention, and long-term adoption of healthy strategies (Evans et al., 2024). Recent intervention-oriented studies show that vocal health education can improve awareness and self-management among professional voice users and music-related populations. Liddell et al. (2026), for example, reported improvements in self-perceived knowledge, confidence, awareness, and vocal health practices following an online vocal health education programme for music therapists.

Lücking and Claßen (2026) found that preventive voice training could support vocal function, vocal self-concept, and wellbeing among university personnel. Diaz et al. (2026) also reported preliminary positive findings on flow phonation exercises for undergraduate singers in training. However, these studies also highlight a key limitation in the existing evidence base: intervention studies show what may work after a programme is introduced, but they do not necessarily explain what undergraduate vocal students need before such support is designed. The present study addresses this preliminary gap by identifying the target and learning needs that should inform future intervention or material development. In this sense, the study does not claim to test a vocal health intervention. Rather, it establishes the needs-based foundation for designing one.

### 4.4 Expert-identified psychological learning needs related to vocal health

A key finding of this study is that experts identified psychological learning needs related to vocal health. Experts linked vocal problems with anxiety, nervousness, low confidence, motivation loss, emotional tension, and reluctance to perform. These accounts suggest that vocal health education may need to address emotional readiness, confidence-building, relaxation, and performance preparation. However, these findings should not be interpreted as direct empirical evidence of students’ psychological states or psychological processes. Rather, they represent expert-informed perceptions of the psychological learning needs that may be relevant to vocal health self-regulation in undergraduate vocal music education. This expert-informed interpretation aligns with Hu (2024), who found that self-efficacy and motivation were negatively associated with music performance anxiety, and with Maoqing (2026), who showed that self-efficacy, self-worth, and intrinsic motivation mediated the relationship between social support and performance anxiety among vocal music students. Recent *Frontiers in Psychology* research further supports this interpretation. Huawei and Jenatabadi (2024) found that emotional intelligence and self-efficacy mediated the relationship between social support and music performance anxiety among Chinese university music students.

Similarly, Ou and Qin (2025) showed that music learning self-efficacy and music performance self-efficacy may relate differently to music performance anxiety among undergraduate violin students in mainland China, suggesting that psychological readiness in music learning should be examined with attention to the specific forms of confidence required for learning and performance.

The relevance of these psychological learning needs is particularly strong in vocal music education. Pan et al. (2025), in their re-validation of the Kenny Music Performance Anxiety Inventory-revised among Chinese university students majoring in vocal music, emphasized that vocal performers are highly exposed to social evaluation because they rely on the body as the direct medium of vocalization and emotional communication. Their findings suggest that music performance anxiety among vocal students involves not only performance pressure, but also emotional regulation difficulties, insecurity, negative self-evaluation, and vulnerability to self-denial. This is closely aligned with the present study, where experts described vocal health problems as connected to anxiety, confidence, motivation, and students’ willingness to continue performing.

The present results also align with Peistaraite and Clark’s (2020) argument that emotion regulation can support self-regulated learning among musicians. Bi and Rauduvaitë (2025) similarly showed that self-efficacy development in singing-related coursework is associated with confidence, emotional regulation, collaboration, adaptability, targeted feedback, and emotionally safe learning environments. These findings support the view that vocal health education should not focus only on the vocal mechanism. If anxiety, rumination, suppression, or performance pressure affects practice and performance, then vocal health education should include confidence-building, emotional regulation, relaxation, reflective awareness, and performance-readiness strategies.

This interpretation is also supported by intervention-oriented evidence. Cong (2026) found that music psychology-based vocal performance anxiety management strategies reduced vocal performance anxiety and improved performance quality, psychological resilience, and heart-rate variability among vocal students, with effects sustained at a 3-month follow-up. Although the present study did not test an intervention, such evidence reinforces the need to treat vocal health self-regulation as a psychological and pedagogical learning process. This is especially important because the voice is both a bodily instrument and a personal expressive medium. Damage to the voice may therefore be experienced not only as a technical problem, but also as a threat to identity, confidence, emotional readiness, and artistic participation.

### 4.5 Needs analysis as a critical basis for instructional support

The use of needs analysis allowed this study to move beyond a general claim that vocal health is important. It clarified what is necessary, what is lacking, what experts want, and what learning support may be needed. Vocal health was identified as a necessity because experts described it as foundational to learning, performance, and career development. It was identified as a lack because textbook materials, literature sources, and expert accounts indicated insufficient systematic vocal health support. It was identified as a want because experts recommended practical, visual, and structured materials. Finally, it was identified as a learning need because students require pedagogical support to develop self-monitoring, practice regulation, emotional readiness, and sustainable vocal habits. This supports the broader view that needs analysis is useful for informing instructional design because it identifies gaps between existing conditions and desired learning outcomes (Gould et al., 2004; Mat et al., 2025).

However, the present study also shows that needs analysis can function as more than a preliminary design step. It can operate as a critical interpretive tool for identifying how a learning problem is constructed across materials, literature, and expert practice. This is important for future vocal health education. Without needs analysis, future materials may become content-heavy but behaviourally weak. They may provide information about anatomy, hygiene, or vocal disorders without helping students develop the habits, judgement, confidence, and self-monitoring routines needed to protect the voice in real practice contexts. A need-based approach therefore helps prevent future instructional materials from being merely informational. It pushes vocal health education toward learner-responsive, practice-based, and psychologically informed support.

### 4.6 Pedagogical implications

The results suggest several implications for undergraduate vocal music education. First, vocal health should be integrated into technical instruction. Rather than teaching vocal health as a separate topic, teachers can connect it directly to breath control, resonance, repertoire preparation, warm-up routines, and practice planning. This would help students understand that healthy voice use is part of good technique. Second, students need explicit training in self-monitoring. They should be taught to recognize early signs of vocal fatigue, hoarseness, throat discomfort, vocal tension, reduced flexibility, and delayed recovery. Self-monitoring may be supported through reflective prompts, practice journals, vocal health checklists, or daily tracking tools.

Third, vocal health education should include behavioral regulation. Students need practical strategies for managing practice duration, balancing rest and intensity, maintaining hydration, avoiding vocal overuse, warming up appropriately, and seeking medical support when needed. Fourth, vocal health education should include psychological support. Since experts linked vocal problems with anxiety, confidence, motivation, and emotional tension, vocal pedagogy should include relaxation strategies, emotional regulation, confidence-building, and supportive teacher feedback. Fifth, teaching materials should be practical and visual. Experts recommended diagrams, anatomical illustrations, daily care strategies, disease-prevention content, and applied vocal relaxation exercises. Such materials may help students understand abstract physiological concepts more clearly and apply them in daily practice.

### 4.7 Theoretical contribution

The main theoretical contribution of this study is the needs-based concept of vocal health self-regulation as a psychological and pedagogical learning need. This concept brings together vocal health education, self-regulated learning, health behavior, and music psychology, but it should be understood as a conceptual framing derived from document mapping and expert perspectives rather than as an empirically tested psychological model. The study suggests that sustainable vocal learning may depend not only on technical development, but also on students’ capacity to understand vocal health principles, monitor vocal condition, regulate practice behavior, manage emotional readiness, and seek appropriate support. In this sense, the contribution to educational psychology lies in identifying a set of expert-informed psychological learning needs that future research can examine more directly. Future studies could test this conceptual framing by measuring students’ self-efficacy, motivation, anxiety, emotional regulation, self-monitoring, help-seeking behaviors and vocal health outcomes. The contribution is also relevant to music education because it offers a way to connect technical vocal pedagogy with health education and psychological readiness. Rather than treating singing technique and vocal health as separate domains, the study positions them as interdependent dimensions of sustainable vocal development.

### 4.8 Limitations and future research

This study has several limitations. First, the study used a qualitative needs-analysis design based on document mapping and five expert interviews. The results therefore provide depth of professional insight but do not claim statistical generalisability. Second, the document mapping focused on ten selected vocal music textbooks, some of which were identified through expert familiarity and relevance to higher vocal music education. The textbook set was therefore treated as an expert-informed sample of instructional materials rather than a comprehensive representation of all vocal music textbooks used in Chinese higher education.

Third, the study did not include direct curriculum observation, classroom observation, or institutional syllabus analysis. Therefore, it cannot determine whether individual institutions provide additional vocal-health support through separate courses, workshops, studio teaching routines, health-related activities, or informal teacher guidance beyond the analyzed textbooks. Fourth, the study focused on expert perspectives and did not include student interview data, student surveys, or clinical vocal assessment. As a result, the findings should be interpreted as expert-identified needs rather than direct evidence of students lived experiences, psychological states, or vocal health outcomes. Relatedly, the psychological contribution of this study is primarily conceptual and needs-based. The study identifies psychological learning needs through expert accounts, but it does not empirically measure psychological variables or demonstrate psychological processes among undergraduate vocal students.

Fifth, the study did not establish a causal relationship between limited textbook representation and students’ vocal health self-regulation behavior. Future research could examine this relationship through student perspectives, classroom observation, longitudinal tracking, or mixed-method designs. Sixth, although the expert participants were experienced higher vocal music educators, the study did not systematically collect detailed biographical data on each expert’s vocal tradition, genre specialization, voice type, or institutional curriculum context. The findings should therefore not be interpreted as comparing vocal health needs across different vocal traditions or genres. Future research should examine whether vocal health self-regulation needs differ across classical singing, Chinese national singing, popular singing, musical theater, and other vocal performance contexts.

Seventh, the study did not measure the effectiveness of any vocal health intervention. Future research could develop and test instructional tools, self-monitoring forms, or vocal health modules to examine their effects on students’ awareness, self-efficacy, practice behavior, anxiety, and vocal health outcomes. Future research could also examine how digital, online, or AI-supported learning tools might support vocal health self-monitoring and feedback, while remaining attentive to ethical, pedagogical, and contextual limitations. Despite these limitations, the study provides a needs-based foundation for future intervention research. The results indicate that undergraduate vocal students may require structured support not only for vocal technique, but also for vocal health awareness, self-monitoring, behavioral regulation, emotional readiness, help-seeking, and sustainable practice.

## 5 Conclusion

This study conducted a qualitative needs analysis of vocal health self-regulation in undergraduate vocal music education. Drawing on document mapping of ten selected vocal music textbooks and interviews with five vocal music education experts, the findings suggest that vocal health is recognized by experts as important for learning continuity, performance quality, psychological confidence, and long-term vocal development. However, within the selected instructional materials and expert accounts examined in this study, vocal health appeared insufficiently systematized as a structured learning domain. The study identifies vocal health self-regulation as an expert-informed psychological and pedagogical learning need. Undergraduate vocal students may require more than technical training; they may need structured support to understand vocal health principles, monitor vocal condition, regulate practice behavior, manage emotional tension, seek appropriate support, and adopt sustainable vocal care habits. However, these findings should be interpreted as needs-analysis evidence rather than direct empirical evidence of students’ psychological processes. The contribution of this study therefore lies in identifying the psychological, behavioral, and pedagogical learning needs that can inform future vocal health education, instructional material development, and empirical research.

## Data Availability

The de-identified data supporting the conclusions of this article will be made available by the corresponding author upon reasonable request, subject to ethical and participant-confidentiality considerations. Full interview transcripts are not publicly available because they may contain information that could identify participants.
